# Supplementary material for: Bio-Fermented Malic Acid Facilitates the Production of High-Quality Chicken via Enhancing Muscle Antioxidant Capacity of Broilers
Source: Antioxidants (Basel). 2022 Nov 22;11(12):2309. doi: 10.3390/antiox11122309 (PMC9774538; doi:10.3390/antiox11122309)
Supplement: Supplementary file 1 [file antioxidants-11-02309-s001.zip › antioxidants-2035596-supplementary.pdf]

Supplemental Table S1. The list of commercial kits purchased from Nanjing Jiancheng Bioengineering Institute

Index	Name of kits	Cat No.
GSH-Px	Glutathione Peroxidase (GSH-PX) assay kit	A005-1-2
GSH	Reduced glutathione (GSH) assay kit	A006-2-1
SOD	Superoxide Dismutase (SOD) assay kit	A001-3-2
MDA	Malondialdehyde (MDA) assay kit	A003-1-2
T-AOC	Total antioxidant capacity assay kit	A015-1-2
IgA	Immunoglobulin A assay kit	H108-1-2
IgM	Immunoglobulin M assay kit	H109-1-1
IgG	Immunoglobulin G assay kit	H106-1-1
Lactic acid	Lactic Acid assay kit	A019-2-1
